# Impact of joint interactions with humans and social interactions with conspecifics on the risk of zooanthroponotic outbreaks among wildlife populations

**DOI:** 10.1038/s41598-022-15713-6

**Published:** 2022-07-08

**Authors:** Krishna N. Balasubramaniam, Nalina Aiempichitkijkarn, Stefano S. K. Kaburu, Pascal R. Marty, Brianne A. Beisner, Eliza Bliss-Moreau, Malgorzata E. Arlet, Edward Atwill, Brenda McCowan

**Affiliations:** 1 School of Life Sciences, Faculty of Science and Engineering, Anglia Ruskin University, Cambridge, CB1 1PT UK; 2 Department of Population Health and Reproduction, School of Veterinary Medicine (SVM), University of California at Davis, Davis, CA 95616 USA; 3 Animal Behavior Graduate Group, University of California at Davis, Davis, CA 95616 USA; 4 Department of Biomedical Science and Physiology, Faculty of Science and Engineering, University of Wolverhampton, Wolverhampton, WV1 1LY UK; 5 Zoo Zürich, Zürichbergstrasse 221, 8044 Zurich, Switzerland; 6 Animal Resources Division, Yerkes National Primate Research Center, Emory University, Atlanta, GA 30329 USA; 7 Department of Psychology, University of California, Davis, CA 95616 USA; 8 California National Primate Research Center, University of California, Davis, CA 95616 USA; 9 Institute of Human Biology and Evolution, Faculty of Biology, Adam Mickiewicz University, 61614 Poznan, Poland

**Keywords:** Behavioural ecology, Conservation biology, Ecological epidemiology, Evolutionary ecology, Macroecology, Microbial ecology, Urban ecology, Infectious diseases, Social evolution, Biological anthropology

## Abstract

Pandemics caused by pathogens that originate in wildlife highlight the importance of understanding the behavioral ecology of disease outbreaks at human–wildlife interfaces. Specifically, the relative effects of human–wildlife and wildlife-wildlife interactions on disease outbreaks among wildlife populations in urban and peri-urban environments remain unclear. We used social network analysis and epidemiological Susceptible-Infected-Recovered models to simulate zooanthroponotic outbreaks, through wild animals’ joint propensities to co-interact with humans, and their social grooming of conspecifics. On 10 groups of macaques (*Macaca* spp.) in peri-urban environments in Asia, we collected behavioral data using event sampling of human–macaque interactions within the same time and space, and focal sampling of macaques’ social interactions with conspecifics and overall anthropogenic exposure. Model-predicted outbreak sizes were related to structural features of macaques’ networks. For all three species, and for both anthropogenic (co-interactions) and social (grooming) contexts, outbreak sizes were positively correlated to the network centrality of first-infected macaques. Across host species and contexts, the above effects were stronger through macaques’ human co-interaction networks than through their grooming networks, particularly for rhesus and bonnet macaques. Long-tailed macaques appeared to show intraspecific variation in these effects. Our findings suggest that among wildlife in anthropogenically-impacted environments, the structure of their aggregations around anthropogenic factors makes them more vulnerable to zooanthroponotic outbreaks than their social structure. The global features of these networks that influence disease outbreaks, and their underlying socio-ecological covariates, need further investigation. Animals that consistently interact with both humans and their conspecifics are important targets for disease control.

## Introduction

The COVID-19 pandemic has highlighted the importance of understanding infectious disease transmission among wildlife populations at human–wildlife interfaces (HWIs)^1,2^. Global population expansion, and consequential human activities causing aquatic and land-use perturbations, ecotourism, and wildlife trade, have all increased spatial overlap and contact rates between humans and wildlife^3,4^. The resultant HWIs are now widely recognized as ‘hotspots’ for the transmission of zoonotic infectious agents of animal origin that spillover into and spread through human populations from wildlife^5,6^. There are also growing concerns of the inverse effect of zooanthroponotic (infectious agents originating from humans or wildlife that spillback from humans) transmission and consequential outbreaks among wildlife^7,8^. Such risks are especially high in urban and peri-urban environments, where generalist wildlife live in dense populations and frequently encounter humans^9–11^. Nevertheless, there exists little quantitative, and in particular comparative research that unravels cross-population differences, and indeed cross-contextual differences within the same population in terms of the kinds of interactions they experience, in their vulnerability to the spread of zooanthroponotic agents. From an evolutionary perspective, such assessments can provide insights into how infectious disease risk influences, and is in-turn influenced by, (mal)adaptive responses in wildlife socioecology, behavioral flexibility, and risk-taking in such environments^12,13^. From a conservation and public health perspective, they are critical to identify ‘edge’ wildlife, that is individual animals or species ranging at HWIs which can further transmit infectious agents into other wildlife and overlapping humans^9,14^.

Research on disease transmission among wildlife populations at HWIs can be hampered by conceptual and methodological limitations. Traditional research on wildlife populations assumed that the probability of acquiring an infectious agent is equal across individuals within a defined area or cohort^15^. In reality, however, wild animals at HWIs can interact with both other animals and with humans and these interactions vary across individuals, time, and space, forming patterns of associations that could influence infectious agent transmission. Social Network Analysis (SNA), through promising quantitative ways to evaluate animals’ tendencies to interact differently with different socioecological aspects of their environment (e.g., their conspecifics, other overlapping species including humans), offers exciting avenues to capture such associations and their impact on disease transmission^13,16–18^. To date, however, the majority of studies that have implemented SNA to understand disease transmission among wildlife have focused on single behavioral features that define animal-animal interactions (reviewed below, but see^18^ for the construction of primate interspecies networks based on the parasites they share). Some examples of wildlife-wildlife social networks that have been associated with increased risk of infectious agent transmission include shared use of space (e.g. Gidgee skinks, *Egernia stokesii*^19^), contact associations (e.g., giraffes, *Giraffa camelopardalis*^20^), aggression (e.g., meerkats, *Suricata suricatta*^21^), and social grooming (e.g., Japanese macaques, *Macaca fuscata*^22^). Yet, the transmission of zooanthroponotic agents among wildlife at HWIs can be influenced by multiple, potentially interplaying types of interactions. Aside from animals’ interactions with conspecifics, zooanthroponosis within wildlife systems may, for instance, also be influenced by animals’ aggregations around anthropogenic factors, such as contaminated water, soil, human foods, livestock, feral mammals, and indeed humans^9–11^. Among generalist, urban and peri-urban wildlife populations in particular, it is therefore crucial to assess the relative effects of multiple (rather than single or specific) types of interactions, e.g. spatial and social interactions with conspecifics, but also co-occurrence or joint interactions with humans or other anthropogenic factors, on zooanthroponotic transmission and resultant disease outbreaks.

Mathematical models offer critical insights into the occurrence of real-world epidemiological processes^23^. In this regard, network approaches have been extensively combined with compartmental ‘Susceptible-Infected-Recovered (SIR)’ models, that simulate disease transmission and outbreaks by causing entities, such as humans or other animals, to move across ‘susceptible’, ‘infected’, and ‘recovered’ disease states^24,25^. They do so at dynamic probabilities that, based on user specifications of model complexity, can depend on a combination of one or more pathogen-specific epidemiological variables (e.g., transmissibility, basic reproduction number: defined below), host contact patterns (e.g., spatial or social network connectedness), and host attributes (e.g., age-sex class) or intrinsic states (e.g., physiology, rates of recovery). To date, studies that have implemented SIR models in combination with wildlife spatial and social networks have revealed strong associations between network connectedness or centrality of the first-infected individual and simulated zooanthroponotic disease outcomes, such as pathogen extinction times (when all individuals have recovered from the disease and no more individuals can be infected) and outbreak sizes (mean % of infected individuals)^26–28^. Others have implemented cross-species comparative approaches, to reveal associations between simulated disease outcomes and global features of social networks, such as community modularity or clustering, network fragmentation, and network centralization which is the variation or skew in centrality across animals within a group^28,29^. Nevertheless, SIR models are yet to be implemented to understand the relative effects of wildlife social interactions with their conspecifics and their joint aggregations around anthropogenic factors, on disease outcomes.

Human–nonhuman primate interfaces are well-suited to address the above gaps. Beyond sharing close evolutionary histories with humans^30^, several nonhuman primate (hereafter NHP) taxa have shared ecological niche space with humans for long periods of their evolutionary history (e.g., Chacma baboons, *Papio ursinus*, macaques, *Macaca* spp.), or following relatively recent exposure to human activities like ecotourism and habitat encroachment (e.g., chimpanzees, *Pan troglodytes*; mountain gorillas, *Gorilla gorilla beringei*) (reviewed in^31–33^). Unsurprisingly, human–primate interfaces are ‘hotspots’ for inter- and intraspecies disease transmission^32,34,35^. Indeed, NHPs are vulnerable to many diseases contracted from or spilling-back from humans (for example, all African and Asian NHPs are vulnerable to infection from SARS-CoV-2^36^), or act as natural reservoirs of pathogens that can invade and cause epidemics in otherwise uninfected human and wildlife populations.

The genus *Macaca* are among the most ecologically and behaviorally flexible of all nonhuman primates. In the wild, many macaque species, particularly rhesus macaques, long-tailed macaques (*M. fascicularis*), and bonnet macaques (*M. radiata*), are considered ‘edge’ wildlife species that form ‘synanthropic’ associations^37^ with humans across a variety of peri-urban landscapes (e.g. cities, temples, rural fields) where they experience highly spatiotemporally variant overlap with humans^38,39^. Influenced by their ecology and evolutionary history, macaques also show marked variation in social behavior with their conspecifics and (consequently) social networks^40–42^. While zooanthroponotic agents have been extensively documented among macaque populations synanthropic with humans (reviewed in^11^), the pathways that underlie their transmission and consequential disease outbreaks remain unclear.

Across human–macaque interfaces in India and Malaysia, we used network approaches combined with SIR models to evaluate the dynamics of zooanthroponotic transmission and outbreaks among multiple groups and species of macaques living in urban or peri-urban environments. In doing so, we evaluated the relative vulnerability of these wildlife populations to human-induced disease outbreaks, through both their joint aggregations around and co-interactions with humans, and through their social interactions with conspecifics that underlie their social structure. To capture patterns of macaques’ co-interactions with humans, we constructed networks of macaques’ (nodes) tendencies to jointly co-occur around and engage with humans (edges) within the same time and location in the context of anthropogenic spaces^43^. To capture patterns of macaque-macaque social interactions, we constructed social ‘grooming networks’ that linked macaques based on the proportions of time they spent engaging in grooming their conspecifics, given the extensive previous literature on the importance of grooming patterns in defining primate social structure^44,45^. In a previous study, we revealed that macaques’ grooming relationships did not predict their tendencies to co-interact with humans^43^. Through this finding, we established a premise to expect that the patterning and distribution of such co-interactions with humans can be different, somewhat independent pathways for disease transmission than their social structure defined through the patterning and distribution of macaque grooming of conspecifics^43^.

Independent of pathogen ‘transmissibility’ (defined below) from an infected individual to a susceptible individual during its infectious period^28^, we examined the impact of the behavioral ecology of wildlife host species at HWIs on disease transmission and outbreaks. Specifically, we examined the effects of cross-contextual (aggregations around and co-interactions with humans, versus grooming of conspecifics) variation in network structure for a given species, and cross-species (rhesus, long-tailed, and bonnet macaques) variation in network structure within a given context, on disease outbreak sizes as predicted by epidemiological models. Rhesus and long-tailed macaques, compared to bonnet macaques, typically show greater ecological flexibility and overlap with anthropogenic environment^46,47^. In many parts of their range, including in our own study locations, such differences in anthropogenic overlap may also be characterized by greater propensities for animals to form denser aggregations around anthropogenic factors and interactions with humans^47^, thereby leading to more well-connected human co-interaction networks compared to among bonnet macaques. In terms of their social structure, however, rhesus macaques and long-tailed macaques are hypothesized to show greater degrees of nepotism and despotism that, compared to bonnet macaques, may be characterized by grooming networks that may be less well-connected^40–42^. Given these expected similarities and differences in network structure, we tested the following specific predictions.

We first predicted that the connectedness, or (hereafter) centrality, of the first-infected macaque, irrespective of context, group, or species, would be positively correlated to outbreak sizes. Based on expected cross-context and cross-species differences in overall network connectedness and structure, we further predicted that the strength of these effects of individuals’ centrality on outbreak sizes would vary across contexts and across species, while controlling for intraspecific variation. Across contexts for a given species, we predicted that for rhesus and long-tailed macaques, co-interaction networks would lead to greater outbreak sizes compared to grooming networks. That is, the centrality of first-infected individuals would have a stronger impact on outbreak sizes through their co-interaction networks than the centrality of first-infected individuals through their grooming networks. On the other hand, we predicted that bonnet macaques would show the opposite effects: a greater effect of the centrality of first-infected individuals on outbreak sizes through their grooming networks compared to their co-interaction networks. Across host species for a given context, we predicted that for human co-interaction networks, rhesus and long-tailed macaques would show a stronger effect of the centrality of first-infected individuals on outbreak sizes compared to bonnet macaques. On the other hand, we predicted the opposite effects for grooming networks: that bonnet macaques would show a stronger effect of the centrality of first-infected individuals on outbreak sizes compared to rhesus and long-tailed macaques.

Furthermore, following a previous study by other researchers on barbary macaque tourist interactions^26^, we also examined the effects of inter-individual differences in the sociodemographic characteristics (sex, dominance rank) of first-infected macaques on outbreak sizes, through both types of networks. Since females and high-ranking individuals form the core of macaque grooming networks^40,41^, we expected that across species, outbreak sizes through grooming networks would be higher when the first-infected individuals were females (versus males) and higher-ranking (versus lower-ranking) individuals. On the other hand, given the exploratory and increased risk-taking behavior of males resulting in their being more well-connected in co-interaction networks compared to females^43,47^, we expected that outbreak sizes through co-interaction networks would be higher when the first-infected individuals are males (versus females). We also explored whether inter-individual differences in their overall anthropogenic exposure (measured as the total frequencies of interactions with humans, and foraging on anthropogenic foods) impacted model-predicted outbreak sizes through both types of networks.

## Materials and methods

### Study locations and subjects

We observed 10 macaque groups representing three different species at human–primate interfaces across three locations in Asia – four groups of rhesus macaques in Shimla in Northern India (31.05°N, 77.1°E) between July 2016 and February 2018, four groups of long-tailed macaques in Kuala Lumpur in Malaysia (3.3°N, 101°E) between September 2016 and February 2018, and two groups of bonnet macaques in Thenmala in Southern India (8.90°N, 77.10°E) between July 2017 and May 2018. All macaque groups were observed in urban to peri-urban environments, and their home ranges overlapped with humans and anthropogenic settlements—Hindu temples (Shimla and Kuala Lumpur), recreational parks (outskirts of Kuala Lumpur, Thenmala), roadside areas (Thenmala, Shimla)—to varying extents^47–49^. Subjects were adult male and female macaques which were pre-identified during a 2-month preliminary phase prior to data collection at each location. More details regarding the study locations, macaque group compositions and subjects, and observation efforts, can be found in our previous publications^47–49^ and in Supplementary Table 1.

### Data collection

We collected behavioral and demographic data noninvasively using observation protocols that were standardized across observers within and across locations (details in^43,47^). All data were collected for 5 days a week, between 9:00 am and 5:00 pm. To record spatiotemporal variation in human–macaque socio-ecological interactions for the construction of co-interaction networks, we used an *event sampling* procedure^50,51^. For this we divided pre-identified parts of the home range of each macaque group in which human–macaque interactions were most likely to occur, into blocks of roughly equal area and observability. We visited these blocks in a pre-identified, randomized order each day. Within a 10-min sampling period, we recorded interactions between any pre-identified subject macaque and one or more humans that occurred within that block, in a sequential manner. Human–macaque interactions included all contact and non-contact behaviors (e.g., approach, aggression, begging for food, provisioning with food) initiated by macaques towards humans or vice-versa, that occurred within a three-meter radius of each other (more details in^48,49^).

To record macaques’ social behavior, and their overall anthropogenic exposure independent of spatiotemporal context, we used *focal animal sampling*^50^. For this we followed individual subjects in a pre-determined, randomized sequence for 10-min durations. In a continuous manner, we recorded, within each focal session, instances of social grooming, and dyadic agonistic interactions that involved aggression (threat, lunge, chase, attack) that was followed by submission (avoidance, silent bared teeth, flee), between the focal animal and its group conspecifics. We also recorded interactions between the focal animal and one or more humans in a continuous manner (see above for definitions). Once every two minutes, we ceased recording continuous data to conduct a *point-time scan*^50^ of the focal animal’s main activity—one of resting, locomotion, socializing, interacting with a human, foraging on natural food, or foraging on anthropogenic food.

We entered all data into Samsung Galaxy Tablets using customized data forms created in HanDBase® application (DDH software). From these we exported and tabulated all the data into MS Excel and MS Access databases daily. All observers within and across locations passed inter-observer reliability tests using Cohen’s kappa (> 0.85)^52^.

### Construction of co-interaction networks and grooming networks

**Figure 1:**
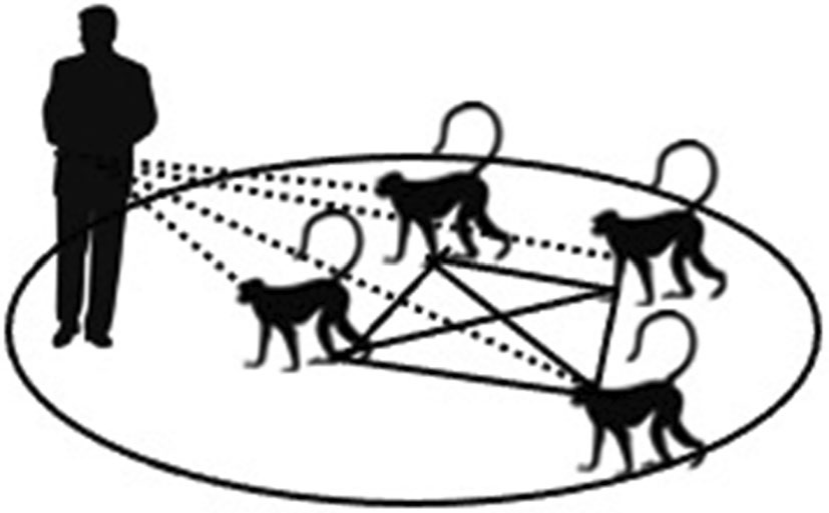
The assignment of edge (solid lines) for macaques’ human co-interaction networks, based on their co-occurrence and joint interactions with humans (dotted lines) within the same time (10-min time-frames) and space (pre-defined blocks of anthropogenic features within the macaques’ home-ranges) (more details in^43^).

From the human–macaque interactions collected using event sampling data, we constructed socio-ecological co-interaction networks. In these, nodes were individual macaques. Edges were based on the frequency with which two or more macaques jointly engaged in at least one interaction with one or more humans at the same block and within the same event sampling session (i.e., a single edge was assigned for each such joint interaction), per unit of event sampling observation time during which both members of the pair were present in the group and (thereby) observable^43^ (Fig. 1). We also constructed macaque-macaque social grooming networks using the focal sampling data. In these, we linked individual macaques (nodes) based on the frequency with which they engaged in social grooming interactions per unit of total focal observation times (edges) calculated for each pair of macaques during the period of their overlapping tenure in the group. Our use of different types of data (event sampling versus focal sampling) to construct co-interaction networks and social grooming networks respectively, reduced the potentially confounding effects of data inter-dependencies and sampling bias on our networks^53^.

### Macaque network centrality, sociodemographic attributes and overall anthropogenic exposure

For each co-interaction network and grooming network, we calculated individual animals' weighted degree or strength centrality, i.e. the sum of all the edge-weights of an individuals’ direct network connections. We used this measure because it generally performs better than other measures of centrality (e.g. unweighted degree, but also betweenness and eigenvector centrality) in predicting both individuals’ risk of infection and group-specific outbreak sizes^54^. Among other wild animal social groups, measures like betweenness and eigenvector centrality that incorporate indirect network connections have been shown to not add any more meaningful information than strength, to the prediction of disease outcomes^27,54^. For each macaque group, we re-scaled strength centrality to range between 0 (least central individual) and 1 (most central individual). Such standardization not only accounts for between-group differences in size, but is also particularly well-suited for the purposes of evaluating cross-network (context-type and species) differences in the effects of the centrality of first-infected macaques on outbreak sizes, as was required here.

From the data on dyadic agonistic interactions with clear winners and losers, we calculated macaques’ dominance ranks for each group, separately for male-male and female-female interactions, using the network-based Percolation and flow-conductance method with a maximum path-length of four steps (Package *Perc* in R^55^). Perc is a network-based ranking method, that has been shown to yield animal rank orders that are consistent with those yielded by other, popularly used ranking methods in behavioral ecology, such as David’s score, I&SI ranks, and Elorating^56,57^. As with network centrality, we converted ordinal ranks of macaques within each group into percentile values that ranged between 0 (lowest-ranked individual) and 1 (highest-ranked individual). We also calculated, for each macaque, its overall frequency of interactions with humans per unit focal observation time, and its time spent foraging on anthropogenic food as the ratio of the number of point-time scans in which it was foraging on anthropogenic food (Fa) to the total number of scans in which it was foraging on either anthropogenic food (Fa) or natural food (Fn): Fa/(Fa + Fn) (more details in^47–49^).

The behaviors used to compute measures of macaques’ overall anthropogenic exposure were also among those used in the constructions of human co-interaction networks described earlier. To reduce inter-dependency issues, we therefore calculated measures of overall anthropogenic exposure from continuously collected focal sampling data, rather than from the event sampling data that was used to construct and calculate co-interaction networks. Measures of overall anthropogenic exposure, in addition to contexts of joint interactions with humans, also captured macaques’ interactions with humans in the absence of conspecifics, as well as their foraging on anthropogenic food outside of contexts in which they were directly provisioned by humans (e.g., from garbage-bins). For these reasons, we anticipated that these measures would, at the most, be weakly correlated with macaques’ centrality within human co-interaction networks (confirmed by collinearity diagnostics performed below).

### Disease simulations

**Figure 2:**
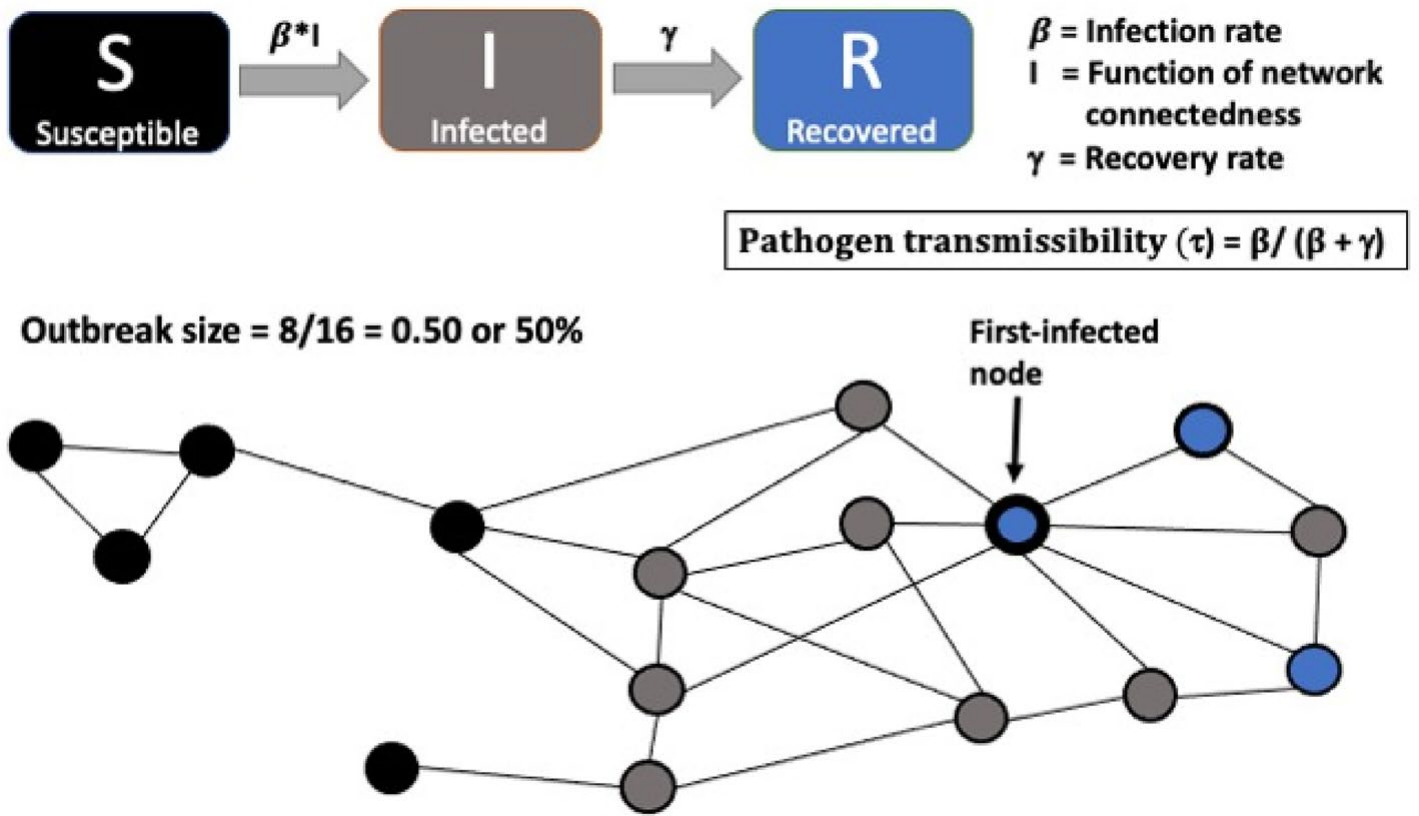
A typical Susceptible-Infected-Recovered (SIR) model simulation of network-mediated disease transmission.

To simulate the spread of zooanthroponotic agents of varying transmissibility (τ) on macaques’ co-interaction networks and grooming networks, we ran a series of SIR models (using the *Epimdr* R package^58^) (Fig. 2). We define ‘τ’ as a pathogen-specific characteristic, i.e. its probability of infecting a susceptible host within its infectious period which is a function of the probability of pathogenic infection ($\beta$) and recovery rate (γ), and is calculated as $\beta /(\beta + \gamma )$^28^. For each network type (human co-interaction, social grooming) and macaque group, we ran 5000 model simulations, 500 for each of 10 different values of τ ranging from 0.05 to 0.50 in increments of 0.05. These selections were based on the human literature that indicates that these values of τ correspond to pathogens that range from low (e.g., influenza virus^59^), to moderate (e.g., respiratory pathogens like SARS-CoV-2^60^), to high (e.g., measles virus^15^) contagiousness, and average basic reproduction numbers (R_0_) of between 1.6–14.0^27,28^. We thus ran a total of 100,000 simulations (5000 per macaque group times 10 groups times two network types). In each simulation, we deemed all macaques within a group to be initially ‘susceptible’, and then infected one individual (node) at random with an artificial pathogen of a given τ. A simulation proceeded using a discrete time, chain binomial method^28,61^ that dynamically and temporally tracked the spread of infection through a weighted, undirected network through time. In each simulation, animals were allowed to transition from ‘susceptible’ to ‘infected’ states, as a function of their network connections to individuals already in ‘infected’ states and the pathogen τ value. ‘Infected’ individuals were then allowed to transition into ‘recovered’ states at a fixed recovery rate (γ) of 0.2 that corresponds to an average infectious period of 5 days^28^. Each simulation was allowed to proceed until the disease proceeded to extinction when there were no remaining infected individuals in the network. At the end of each simulation, we calculated the disease outcome of ‘mean outbreak size’, as the average % of infected macaques (the number of ‘infected’ individuals divided by the total number of individuals) across all time-units of the simulation. We also extracted, for each simulation, the identity of the first-infected macaque (Fig. 2) and calculated an average of outbreak sizes from across all its first-infected simulation runs. We then matched this individual-level mean outbreak size with the sociodemographic characteristics, network centrality, and overall anthropogenic exposure of this (first-infected) individual.

### Statistical analysis

We used General Linear Mixed Models (GLMMs) with a beta error structure (package *glmmTMB*^62^) to test our predictions. In all GLMMs, we set ‘mean outbreak size’, calculated as the proportion of the number of infected individuals divided by the total group size, as the outcome variable. This was calculated at the level of individual macaques, as the average outbreak size across all the simulations in which it was the first-infected individual, for both co-interaction networks and grooming networks. To examine the effects of cross-context differences for a given host species on outbreak sizes, we ran three GLMMs, one model for each macaque species. In these, we included, as main effects, the network strength centrality of the first-infected macaque, network type to define the context (co-interaction versus grooming), and an interaction term of network centrality by network type to determine whether the magnitudes of the effects of network strength on outbreak sizes were different across co-interaction networks and grooming networks. We also included, as main effects, the sociodemographic attributes (sex, dominance rank) and the overall anthropogenic exposure (frequencies of interactions with humans, proportions of time spent foraging on anthropogenic food) of the first-infected macaque. In all three models, we also included a random-effects term of macaques’ strength centrality, dominance rank, frequencies of interactions with humans, and foraging on anthropogenic food as random slopes, all nested within macaque ‘group ID’ as a random intercept, to control for between-group, within-species differences.

To examine the effects of cross-species differences for a given context on outbreak sizes, we ran two more GLMMs, one for each context or network type. In these, we included, as main effects, the strength centrality of the first-infected macaque, its species (bonnet versus long-tailed versus rhesus), and an interaction term of strength centrality by species to determine whether the magnitude of the effect of network strength on disease outbreaks was different across the three species. As in the first three models above, we also included first-infected macaques’ sociodemographic attributes (sex, dominance rank) and overall anthropogenic exposure (frequencies of interactions with humans, proportions of time spent foraging on anthropogenic food) as main effects. Likewise, we also included a random-effects term, of macaques’ strength centrality, dominance rank, frequencies of interactions with humans, and foraging on anthropogenic food as random slopes, nested within macaque ‘group ID’ as a random intercept.

All GLMMs met the necessary assumptions of model validity including the distribution of residuals, residuals plotted against fitted values^63^ and collinearity diagnostics (using the *performance* R package^64^). The latter showed no strong correlations (Pearson’s r < 0.28) between continuous main effects variables (dominance rank, strength centrality within co-interaction networks or grooming networks, overall frequencies of interactions with humans, and proportions of times spent foraging on anthropogenic food). All statistical tests were two-tailed, and we set the p values (extracted from the model outputs) to attain statistical significance to be < 0.05.

### Animal welfare and ethics statement

The protocols used in the study were approved by the Institutional Animal Care and Use Committee (IACUC) of the University of California, Davis (protocol # 20593). The research was performed strictly in accordance with the guidelines and regulations drafted in this protocol. Observers did not engage in any contact or non-contact interactions with the animals while recording their natural behavior. No biological samples were collected. Since exclusively observational data were collected on both the monkeys and humans, with no identifying information collected on the humans and no interactions between the experimenters and the humans, no human subjects were enrolled to directly participate in this study. This protocol, along with the guidelines and regulations, was designed in consultation with the Himachal Pradesh Forest Department and the Indian Institute of Science Education and Research Thiruvananthapuram in India, and Universiti Putra Malaysia and Universiti Sains Malaysia in Malaysia. They complied with the legal requirements of India and Malaysia.

## Results

**Figure 3:**
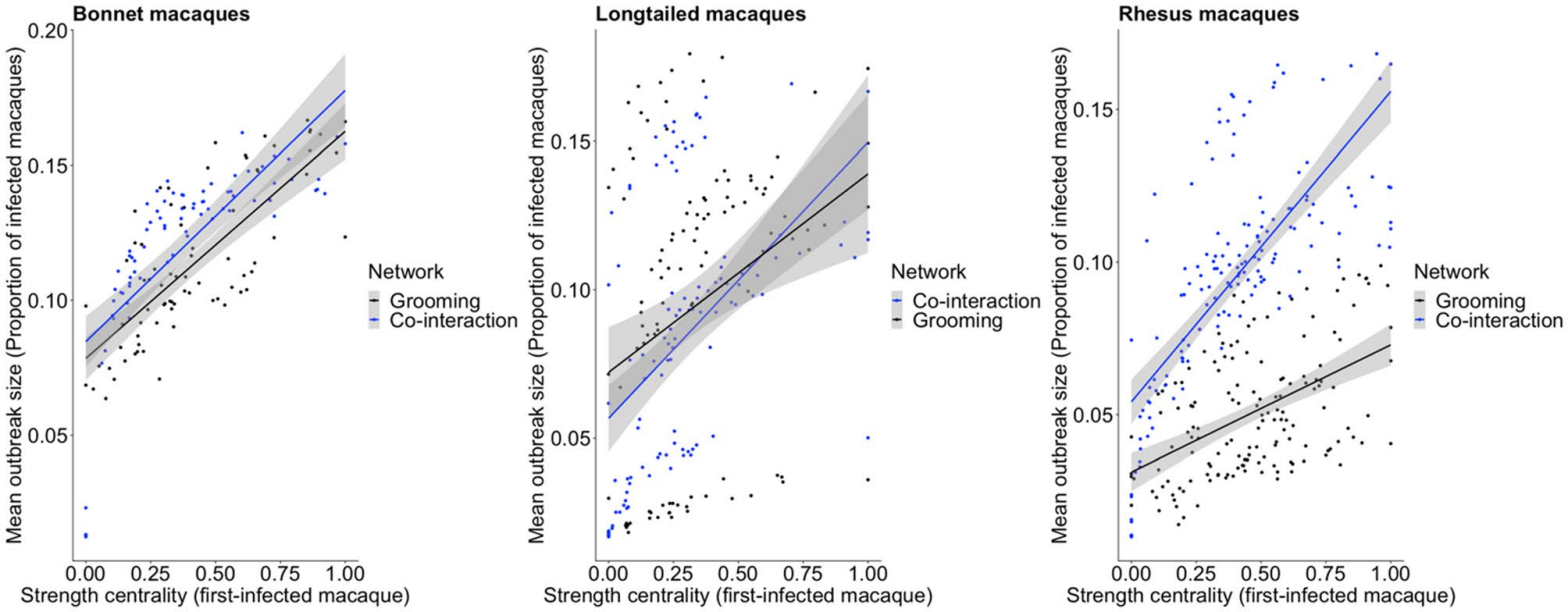
Scatterplots showing positive correlations, and the differences in these correlations (slopes) across contexts, between the strength centrality of first-infected macaques through their human co-interaction networks and their grooming networks, for each host species.

**Figure 4:**
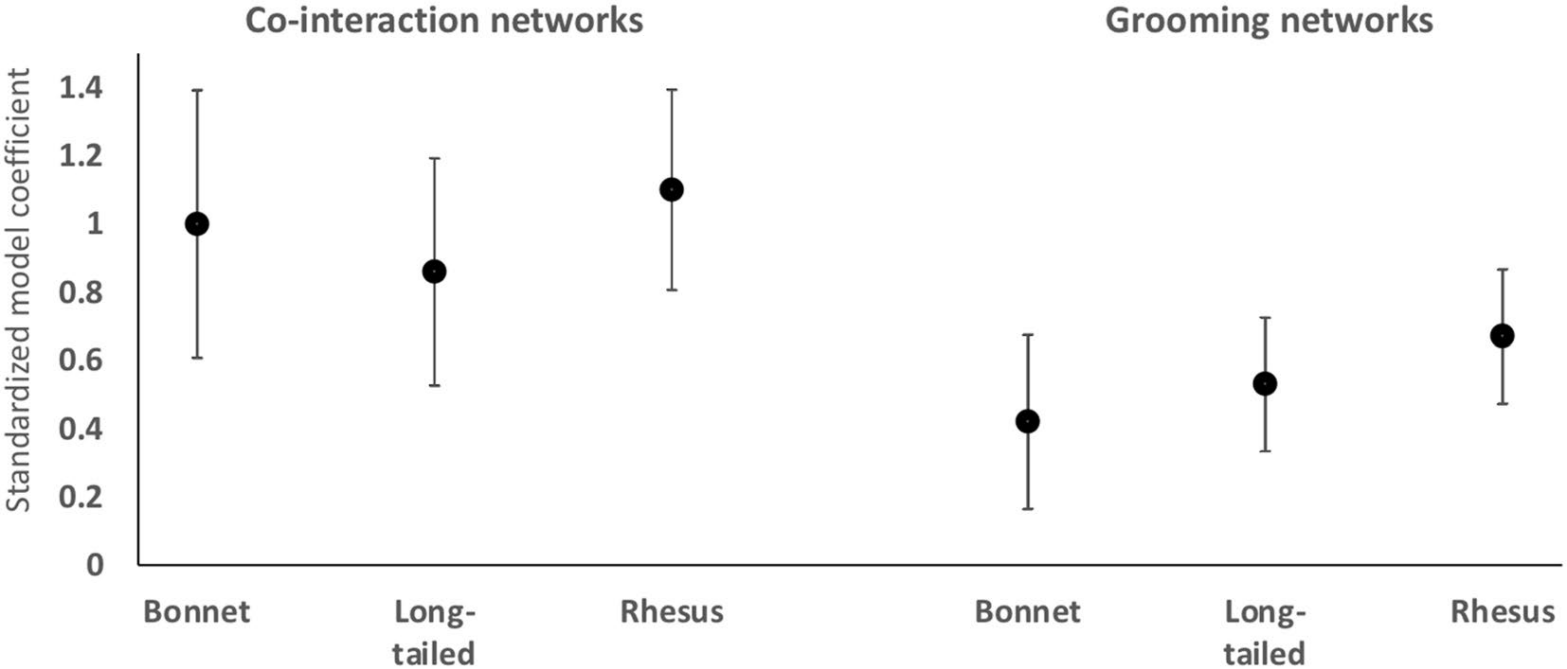
Plots of standardized model-coefficients (Y-axis; values from Table 2) to show the effects of the strength centrality of first-infected macaques on outbreak sizes by species, through co-interaction networks and grooming networks. Error bars represent 95% confidence intervals for each coefficient.

In support of our prediction, we found that independent of context (human co-interactions and grooming) and host species (rhesus, long-tailed, and bonnet macaques), the strength centrality of the first-infected macaque was significantly, positively correlated to mean outbreak size (Tables 1 and 2; Figs. 3 and 4). Moreover, there were cross-contextual differences by species in the effects of network strength centrality of first-infected macaques on outbreak sizes. However, these differences were not always in the predicted directions.

**Table 1:** Standardized model coefficients from the GLMMs (full model parameters in Supplementary Table 2) that examined the effects of context (co-interaction vs grooming), network strength centrality of the first-infected macaque, and the interaction between context and centrality, for each macaque (host) species.

Predictor	Model coefficients		
	Bonnet macaques	Long-tailed macaques	Rhesus macaques
(Intercept)	− 2.45**	− 2.72**	− 2.71**
Sex (males vs females)	− 0.11*	− 0.01	− 0.05
Rank percentile	0.07	0.07	0.06
Context (grooming vs co-interaction)	− 0.19*	0.11	− 0.46**
Network strength (co-interaction)	1.01**	0.59*	1.12**
Network strength (grooming)	0.42**	0.52*	0.66**
Frequency of interactions with humans	0.10	0.08	0.02
Foraging on anthropogenic food	0.12	0.08	− 0.05
Network strength by context (grooming vs co-interaction)	− 0.59**	0.06	− 0.45**

*p < 0.05; **p < 0.01.

**Table 2:** Standardized model coefficients from the GLMMs (full model parameters in Supplementary Table 3) that examined the effects of macaque species (rhesus vs long-tailed vs bonnet), network strength centrality of the first-infected macaque, and the interaction between species and centrality, for each context.

Predictor	Model coefficients	
	Co-interaction	Grooming
(Intercept)	− 2.45**	− 2.20
Sex (males vs females)	− 0.05	− 0.08*
Rank percentile	0.04	0.08*
Species (long-tailed vs bonnet)	− 0.31	− 0.39
Species (rhesus vs bonnet)	− 0.27	− 1.05*
Species (long-tailed vs rhesus)	− 0.04	0.65
Network strength (bonnet)	1.00**	0.42**
Network strength (long-tailed)	0.86**	0.53**
Network strength (rhesus)	1.10**	0.67**
Frequency of human–macaque interactions	0.05	0.03
Foraging on anthropogenic food	0.06	0.02
Network strength by species (long-tailed vs bonnet)	− 0.14	0.11
Network strength by species (rhesus vs bonnet)	0.10	0.25
Network strength by species (long-tailed vs rhesus)	− 0.24	− 0.14

*p < 0.05; **p < 0.01.

Across different contexts for a given host species, we found a significant interaction between context and network strength centrality for rhesus macaques and bonnet macaques, but not for long-tailed macaques (Table 1; Fig. 3). Exploring this interaction further revealed that, for all three species, the centrality of first-infected macaques within their human co-interaction networks had a stronger effect on outbreak sizes than the centrality of first-infected macaques within their grooming networks. As predicted, rhesus macaques showed a significantly stronger effect of network centrality of first-infected individuals on outbreak sizes through their co-interaction networks compared to their grooming networks (Table 1; Fig. 3). Contrary to our predictions, bonnet macaques also showed the same (rather than the opposite) effect as rhesus macaques (Table 1; Fig. 3). For long-tailed macaques, the centrality of first-infected macaques within their co-interaction networks had a marginally greater effect on outbreak sizes than the centrality of first-infected macaques within their grooming networks, but this difference was not significant (Table 1; Fig. 3). Moreover, long-tailed macaques also seemed to show separate groupings within each network type (Fig. 3), suggesting possible intraspecific differences in the effects of co-interaction and grooming network structure on outbreak sizes (see Discussion).

Across host species in a given context, we found no significant interactions between species and network strength centrality, for both co-interaction networks and grooming networks (Table 2). That is, co-interaction network centrality showed a similar, and significantly positive, effect on outbreak sizes across all three species (Table 2; Fig. 4). Likewise, the effect of grooming network centrality on outbreak sizes was also similar, and significantly positive, across all three species (Table 2; Fig. 4). Nevertheless, for all three species, the magnitudes of these effects of strength centrality on outbreak sizes were markedly greater for co-interaction networks compared to grooming networks (Fig. 4). In other words, across host species, the centrality of macaques within their co-interaction networks led to consistently higher outbreak sizes (greater proportions of individuals infected) than the centrality of macaques within their grooming networks (Fig. 4).

For grooming networks, but not for co-interaction networks, we also found a significant effect of sex and dominance rank of the first-infected individual on mean outbreak sizes—outbreak sizes were higher when first-infected macaques within grooming networks were females compared to males, and higher-ranking compared to lower-ranking individuals (Table 2). However, these effects were of much lower magnitude than those of the strength centrality of first-infected macaques (Table 2). Moreover, the effect of sex seemed to be largely driven by a single species, given that bonnet macaques, but not rhesus macaques or long-tailed macaques, showed a significant effect of sex (females > males) on mean outbreak sizes (Table 1). Finally, the overall anthropogenic exposure of first-infected macaques, specifically their frequencies of interactions with humans and times spent foraging on human foods, had no impact on outbreak sizes (Tables 1, 2).

## Discussion

We addressed a critical gap in our understanding of disease ecology at HWIs, by showing cross-contextual and cross-species differences in the vulnerability of wildlife populations living in peri-urban environments to zooanthroponotic outbreaks. Moreover, our approaches built on previous comparative studies of disease outbreaks through wildlife behavioral networks (e.g.^28,29^) in some important ways; we evaluated both cross-contextual and cross-species differences in network-mediated outbreak sizes, and did so using behavioral datasets that were collected using sampling methods that were identically implemented across populations.

In all three macaque species, we found that outbreak sizes were positively predicted by the centrality of first-infected macaques within both their co-interaction networks and their grooming networks. That is, zooanthroponotic transmission and outbreaks were influenced by the connectedness and emergent network structure of macaques’ joint aggregations around humans and their social interactions with conspecifics. By comparing the relative risk of disease outbreaks posed by animals’ joint interactions with humans with that posed by their interactions with conspecifics, we build on previous, network-based studies that have focused on modelling disease outbreaks among wildlife populations through animal social structure alone (e.g. European badgers, *Meles meles*^54^; chimpanzees^27^; barbary macaques, *Macaca sylvanus*^26^; interspecies comparative studies^28^). Among the most widespread, ecologically flexible of all mammals outside of the family Rodentia, wild macaques, in many parts of their range, live in dense populations in a variety of anthropogenic environments (e.g., urban, agricultural, forest-fragmented habitats), where they frequently interact with humans. From socio-ecological perspectives, our findings should therefore encourage future studies on other, group-living wildlife populations that better distinguish between, and evaluate the relative effects of, wildlife co-occurrence and interactions with anthropogenic factors and their social behaviour towards conspecifics, on disease transmission and outbreaks (e.g. elephants, *Loxodonta africana*, in agricultural fields^65^; co-occurrence and space-use sharing of wild ungulates and livestock^66^; human provisioning of birds and raccoons, *Procyon lotor*, in urban environments^10^).

We found cross-contextual differences in the effects of network centrality on outbreak sizes, but not always in the predicted directions. Specifically, the centrality of macaques within their co-interaction networks consistently led to higher outbreak sizes compared to their centrality within grooming networks. In other words, zooanthroponotic agents, following a spillback event from a human to a macaque, can spread faster among macaques on account of the structural features of animals’ tendencies to congregate around anthropogenic factors, than on account of the distribution of grooming interactions that underlie their social structure. More generally, the congregations of generalist wild animals around humans and anthropogenic features can lead to an even greater vulnerability of wildlife populations to disease outbreaks than their social interactions and emergent social structures.

For a given context, there were no cross-species differences in the effect of network centrality on outbreak sizes. Nevertheless, we found that the relative degree or extent of cross-contextual differences in the effects of network centrality on outbreak sizes varied across species. As predicted, rhesus macaques were more vulnerable to disease outbreaks through co-interaction networks, and less vulnerable through their grooming networks. One reason for this could be the typically high degrees of preference towards affiliating with close kin in this species (called grooming kin bias^67^), that may also underlie greater sub-grouping within rhesus macaque grooming networks^40–42^. Such sub-grouping, while facilitating pathogen transmission within clusters, may also inhibit transmission between sub-groups, and thereby the sizes of group-wide outbreaks^29,68^. Yet animals that show sub-divided social networks can also be vulnerable to outbreaks through other types of associations, and often in specific socio-ecological contexts around human-provisioned food that may cause wild animals to aggregate together^10,69^ and co-interact with humans. In rhesus macaques, greater connectedness and other emergent properties of co-interaction networks seem to more likely facilitate rather than inhibit zooanthroponotic transmission. Corroborating these explanations await more in-depth socio-ecological investigations of the relative effects of macaques’ kin structures and the distribution of anthropogenic food on the extent of sub-grouping (and indeed other global network features discussed further below) within their networks, and their consequential effects on disease outbreaks.

Contrary to our predictions the effects of co-interaction networks on outbreak sizes in bonnet macaques were significantly greater (rather than lesser) than the effects of grooming networks, and were in fact within the range of rhesus macaques. One reason for this may be the spatial distribution of human–wildlife interactions in this population. Bonnet macaques are less geographically widespread and ecologically flexible compared to rhesus macaques^46^. Although the bonnet macaques in our study experienced markedly lower frequencies of interactions with humans compared to rhesus macaques and long-tailed macaques^41^, these interactions were highly geospatially restricted to within specific areas or ‘blocks’ within their home range. It is likely that such spatially dense socio-ecological associations with humans, through increasing the connectivity of macaques within their co-interaction networks, leads to a considerable increase in the risk of disease outbreaks despite their relatively lower overall frequencies of interactions with humans. More generally, this finding suggests that zooanthroponotic agents may enter into and rapidly spread even through populations of less ecologically flexible wildlife that, despite interacting less frequently with humans, have tendencies to congregate around anthropogenic factors within specific parts of their home range (e.g., contexts of food provisioning^67^; crop-foraging^65^; ecotourism activity^26^).

Contrary to our prediction, long-tailed macaques showed no differences in outbreak sizes across network types. At least one explanation for this may be intraspecific variation, which seems to be supported by the separate groupings for the relationships between individuals’ network centrality and outbreak sizes for long-tailed macaques, even for the same network type (Fig. 3). Such intraspecific variation in network structure and resultant outbreak sizes may arise from between-group differences in their overall exposure to anthropogenic factors. We observed two groups of long-tailed macaques at a Hindu temple and popular tourist location within Kuala Lumpur, where the monkeys were exposed to dense human populations with whom they interacted highly frequently^48^. On the other hand, we observed two other groups in at a recreational park at the edge of the city bordering a fragmented forest area, where interactions with humans were comparatively less frequent^48^. Moreover, long-tailed macaques also showed marked differences in their grooming behavior across these locations as a response to interactions with humans^48^, which may underlie differences in their grooming network structure. A more comprehensive assessment of the disease vulnerability of these populations would therefore require within-species, cross-group comparisons of network structure, and the socio-ecological factors that underlie them.

In many wildlife species, animals’ sociodemographic attributes like their sex and dominance rank influence their life-history, behavioral strategies, and adaptive responses to changing (anthropogenic) environments^47,65^. It was therefore important to evaluate the effects of such factors on disease outbreaks. As predicted, outbreak sizes through macaques’ grooming networks were generally higher when the first-infected individuals were females compared to when they were males, or when the first-infected individuals were higher-ranking compared to lower-ranking. Nevertheless these effects, despite reaching statistical significance, were a lot weaker compared to effects of individuals’ network centrality, suggesting that outbreaks are influenced more directly by network connectedness as such rather by animal attributes such as sex and rank that might influence outbreaks through such connectedness^26^. Furthermore, macaques’ overall frequencies of interactions with humans and foraging on anthropogenic food showed no effects on outbreak sizes. In general, individual animals that interact more frequently with anthropogenic factors may be the focal points of zooanthroponotic spillback events^7,8^. However, the likelihood of such spillback developing into group-wide outbreaks would seem to depend more on the extent to which macaques’ interactions with anthropogenic factors are isolated events, versus occur systematically across time and space with multiple (rather than single) animals involved. Our construction of human co-interaction networks effectively captured the latter type of effect, and thereby more clearly predicted outbreak sizes than animals’ overall anthropogenic exposure.

Our findings have implications for conservation, and in particular One Health approaches^5,70^. To date, research on disease transmission through wildlife populations has identified ‘superspreaders’^71^ of pathogens that, in lieu of being more well-connected to other individuals and populations, can function as effective targets for disease control (e.g. vaccination, antimicrobial treatment^16,27,71^). Our findings suggest that macaques which are central in their human co-interaction networks can be especially effective targets, since these individuals can both function as intraspecies superspreaders, as well as pose a high risk of interspecies (humans-to-macaques, or vice-versa) disease transmission events since they inter-connect humans with whom they interact within and across time and space. Confirmation of this awaits future studies that implement multi-modal networks, in which we include pre-identified individual wild animals, but also anthropogenic factors (individual humans, livestock, feral mammals) as nodes that are interlinked based on spatial and/or social interactions^13^. These would enable quantitative risk-assessments of disease spillover (wildlife → humans) and spillback (humans → wildlife)^7,8^, which are of utmost importance for preventing or controlling future epidemics and pandemics^1,32^. They also await studies that simulate pathogen transmission along with targeted disease control interventions (e.g. vaccination, treatment) of select-animals within specific macaque groups and network types based on the results from this studies (as in^27^).

## Limitations and considerations

Our approaches had important limitations and considerations. First, comparisons of disease outbreaks across different behavioral networks often make the underlying assumption that different behaviors would manifest in equal likelihoods of contact^16,28^. However, this is not always the case. For instance, joint interactions with humans would mean that macaques, while within three meters of each other, may or may not engage in direct physical contact as they would while grooming. In that regard, social proximity to their conspecifics would be more comparable (than grooming) with co-interactions with humans. However, proximity captures patterns of affinitive, rather than affiliative (as is the case with grooming in nonhuman primates), interactions between animals that may or may not underlie meaningful social structure^72^. Moreover, our previous work showed strong correlations between proximity networks and human co-interaction networks^43^. Thus, our choice of comparing grooming rather than proximity to co-interactions with humans more readily catered towards our goal of assessing the effects of clear, cross-contextual (anthropogenic aggregations, versus social structure) differences in animals’ overall vulnerability to outbreaks rather than the relative likelihoods of dyadic, distance-based transmission events.

Second, we examined the effects of rescaled, rather than the absolute, values of animals’ network centrality on outbreak sizes. This approach was suitable for our objective of examining variation in slopes, specifically cross-contextual and cross-species differences in the effects of centrality and emergent network structures on outbreak sizes that were calculated through weighted, undirected networks. Moreover, rescaling was necessary to account for between-group differences in group size. Nevertheless, we fell short of examining which global structural feature(s) of networks, e.g. average or mean centrality across individuals^28^, but also the degree of sub-group formation or community modularity^29,73^, or the efficiency of information flow^73^, were the most likely to influence outbreak sizes. Such assessments would be necessary in order to further corroborate the possible socio-ecological explanations for our findings offered earlier. Given our sample size of just 10 macaque groups nested within three species, a more robust assessment of the links between socio-ecological factors, global network measures, and disease outcomes might look to focus on intraspecific, temporal variation among macaque groups. These would look to dynamically track changes to individual behavior and resultant global network features that influence, and are in turn influenced by, disease outcomes, across epidemiologically relevant time-frames that reflect real-world pathogen progression (as in^27^).

Third, our results were independent of pathogen-specific transmissibility which, through influencing basic reproduction numbers (R_0_ values), can strongly impact disease outbreaks. We chose to account for, rather than quantitatively evaluate, the effects of a suite of respiratory pathogens of different transmissibility based on the human literature (e.g., influenza virus, measles virus, *Mycobacterium* spp.), that typically spread through social interactions, might enter wildlife populations from human or livestock carriers and are capable of causing disease in primates^27^. Pathogen transmissibility may interact with animal ecology in complicated ways to influence outbreak sizes. For instance, the effects of animal social interactions on disease outbreaks can diminish for pathogens of exceptionally high transmissibility, which can reach high outbreak sizes irrespective of social connections^27,28^. Yet other studies have revealed that social interactions have stronger effects on outbreak sizes for pathogens of intermediate compared to low or high transmissibility^54^. Given the current lack of disease parameters on these macaque populations, our pathogen transmissibility values were also based on the human epidemiological literature (similar to other epidemiological studies on wildlife populations reviewed above). Inter-host and inter-pathogen differences would need to be considered in future studies that construct more sophisticated but system-specific epidemiological models.

## Supplementary Information

Supplementary Tables.

## Data Availability

The final dataset used to run the GLMMs in this study is now available via figshare: 10.6084/m9.figshare.19539004.v3. The raw edgelists used to construct social networks is currently being used to conduct follow-up analyses pertaining to other, on-going studies that we are conducting. Therefore, we have not published the edgelists with this study, but anticipate doing so in the future. Until then, the edgelists will be made available on request (with the first author).
