# Supplementary material for: Impact of joint interactions with humans and social interactions with conspecifics on the risk of zooanthroponotic outbreaks among wildlife populations
Source: Sci Rep. 2022 Jul 8;12:11600. doi: 10.1038/s41598-022-15713-6 (PMC9263808; doi:10.1038/s41598-022-15713-6)
Supplement: Supplementary file 1 — Supplementary Tables. [file 41598_2022_15713_MOESM1_ESM.docx]

Supplementary Table 1: Information on the study groups, subjects, and observation effort (table borrowed from our previous publication: Marty et al. 2019b)

| **Species** | **Group ID** | **Adult Males** | **Adult Females** | **Network nodes** | **Observation period** | **Focal Observation Hours**  **(mean ± Std. dev.)** |
| --- | --- | --- | --- | --- | --- | --- |
| Rhesus macaques  (*Macaca mulatta*) | RM_G1 | 9 | 18 | 27 | Jun 2016 – Feb 2018 | 11.01 ± 3.14 |
|  | RM_G2 | 7 | 17 | 24 | Jun 2016 – Feb 2018 | 14.46 ± 2.63 |
|  | RM_G3 | 13 | 28 | 41 | Jun 2016 – Feb 2018 | 16.46 ± 5.74 |
|  | RM_G4 | 14 | 45 | 59 | Jun 2016 – Feb 2018 | 8.50 ± 2.65 |
| Long-tailed macaques  (*Macaca fascicularis*) | LM_G1 | 11 | 24 | 35 | Sep 2016 – Feb 2018 | 13.43 ± 4.29 |
|  | LM_G2 | 7 | 12 | 19 | Sep 2016 – Feb 2018 | 13.5 ± 2.88 |
|  | LM_G3 | 15 | 19 | 34 | Sep 2016 – Feb 2018 | 6.98 ± 1.47 |
|  | LM_G4 | 5 | 19 | 24 | Sep 2016 – Feb 2018 | 12.16 ± 2.99 |
| Bonnet macaques  (*Macaca radiata*) | BM_G1 | 26 | 22 | 48 | Jul 2017 – May 2018 | 11.78 ± 2.70 |
|  | BM_G2 | 10 | 18 | 28 | Jul 2017 – May 2018 | 11.42 ± 1.41 |

Supplementary Table 2: GLMMs to examine the effects of variation in network connectedness by context or network type (co-interactions with humans versus grooming) for a given host species on disease outbreaks. For each macaque species, these models examined the effects of network-type, as well as an interaction between the network strength centrality of the first-infected macaque and network-type, on mean outbreak sizes. The latter was calculated at the level of the individual macaque, as the average of the proportion of infected individuals within its group at the end of all the simulation runs in which that individual was the first-infected macaque. In each model, we also included the sociodemographic attributes (sex, dominance rank) and the overall exposure to anthropogenic factors (frequencies of interactions with humans and foraging on anthropogenic food) of first-infected macaques as main-effects. As random effects, we included all the continuous main-effects predictors as random slopes. nested within macaque ‘Group ID’ as a random intercept.

| **Bonnet macaques** | | | | | |  |
| --- | --- | --- | --- | --- | --- | --- |
| **Predictor** | **B** | **SE** | **Z** | **p** | ***df*** | |
| (Intercept) | -2.45 | 0.12 | -20.82 | <0.01** | 138 | |
| Sex (males vs females) | -0.11 | 0.05 | -2.33 | 0.02* |  | |
| Rank percentile | 0.07 | 0.08 | 0.90 | 0.37 |  | |
| Context or network-type (grooming vs co-interaction) | -0.19 | 0.08 | -2.34 | 0.02* |  | |
| Network strength (co-interaction) | 1.01 | 0.10 | 9.69 | <0.01** |  | |
| Network strength (grooming) | 0.42 | 0.14 | 3.09 | <0.01** |  | |
| Frequency of interactions with humans | 0.10 | 0.10 | 1.02 | 0.31 |  | |
| Foraging on anthropogenic food | 0.12 | 0.10 | 1.22 | 0.22 |  | |
| Network strength by context or network-type (grooming vs co-interaction) | -0.59 | 0.16 | -3.64 | <0.01** |  | |

**p < 0.01; *p </= 0.05

| **Long-tailed macaques** | | | | | |  |
| --- | --- | --- | --- | --- | --- | --- |
| **Predictor** | **B** | **SE** | **Z** | **p** | ***df*** | |
| (Intercept) | -2.72 | 0.32 | -8.59 | <0.01** | 210 | |
| Sex (males vs females) | -0.01 | 0.05 | -0.24 | 0.81 |  | |
| Rank percentile | 0.07 | 0.07 | 0.92 | 0.36 |  | |
| Context or network-type (grooming vs co-interaction) | 0.11 | 0.07 | 1.50 | 0.13 |  | |
| Network strength (co-interaction) | 0.59 | 0.23 | 2.54 | 0.01* |  | |
| Network strength (grooming) | 0.52 | 0.24 | 2.21 | 0.03* |  | |
| Frequency of interactions with humans | 0.08 | 0.09 | 0.91 | 0.36 |  | |
| Foraging on anthropogenic food | 0.08 | 0.12 | 0.60 | 0.55 |  | |
| Network strength by context or network-type (grooming vs co-interaction) | 0.06 | 0.18 | 0.35 | 0.72 |  | |

**p < 0.01; *p </= 0.05

| **Rhesus macaques** | | | | | |  |
| --- | --- | --- | --- | --- | --- | --- |
| **Predictor** | **B** | **SE** | **z** | **p** | ***df*** | |
| (Intercept) | -2.71 | 0.17 | -16.35 | <0.01** | 288 | |
| Sex (males vs females) | -0.05 | 0.04 | -1.20 | 0.23 |  | |
| Rank percentile | 0.06 | 0.06 | 1.00 | 0.32 |  | |
| Context or network-type (grooming vs co-interaction) | -0.46 | 0.07 | -6.59 | <0.01** |  | |
| Network strength (co-interaction) | 1.12 | 0.10 | 10.89 | <0.01** |  | |
| Network strength (grooming) | 0.66 | 0.13 | 5.22 | <0.01** |  | |
| Frequency of interactions with humans | 0.02 | 0.08 | 0.21 | 0.83 |  | |
| Foraging on anthropogenic food | -0.05 | 0.07 | -0.66 | 0.51 |  | |
| Network strength by context or network-type (grooming vs co-interaction) | -0.45 | 0.13 | -3.60 | <0.01** |  | |

**p < 0.01; *p </= 0.05

Supplementary Table 3: GLMMs to examine the effects of variation in network connectedness by host species (rhesus versus long-tailed versus bonnet macaques) for a given context or network-type on disease outbreaks. For each network type, i.e. co-interactions with humans and grooming of conspecifics, these models examined the effects of species, as well as an interaction between the network strength centrality of the first-infected macaque and species, on mean outbreak sizes. The latter was calculated at the level of the individual macaque, as the average of the proportion of infected individuals within its group at the end of all the simulation runs in which that individual was the first-infected macaque. In each model, we also included the sociodemographic attributes (sex, dominance rank) and the overall exposure to anthropogenic factors (frequencies of interactions with humans, and foraging on anthropogenic food) of first-infected macaques as main-effects. As random effects, we included all the continuous main-effects predictors as random slopes. nested within macaque ‘Group ID’ as a random intercept.

| **Human co-interaction networks** | | | | | |  |
| --- | --- | --- | --- | --- | --- | --- |
| **Predictor** | **B** | **SE** | **z** | **p** | ***df*** | |
| (Intercept) | -2.45 | 0.29 | -8.32 | <0.01** | 323 | |
| Sex (males vs females) | -0.05 | 0.04 | -1.36 | 0.17 |  | |
| Rank percentile | 0.04 | 0.06 | 0.64 | 0.53 |  | |
| Species (long-tailed vs bonnet) | -0.31 | 0.36 | -0.85 | 0.40 |  | |
| Species (rhesus vs bonnet) | -0.27 | 0.36 | -0.74 | 0.46 |  | |
| Species (long-tailed vs rhesus) | -0.04 | 0.30 | -0.13 | 0.90 |  | |
| Network strength (bonnet) | 1.00 | 0.20 | 4.92 | <0.01** |  | |
| Network strength (long-tailed) | 0.86 | 0.17 | 5.03 | <0.01** |  | |
| Network strength (rhesus) | 1.10 | 0.15 | 7.47 | <0.01** |  | |
| Frequency of interactions with humans | 0.05 | 0.09 | 0.56 | 0.57 |  | |
| Foraging on anthropogenic food | 0.06 | 0.09 | 0.70 | 0.48 |  | |
| Network strength by species (long-tailed vs bonnet) | -0.14 | 0.26 | -0.55 | 0.58 |  | |
| Network strength by species (rhesus vs bonnet) | 0.10 | 0.25 | 0.39 | 0.69 |  | |
| Network strength by species (long-tailed vs rhesus) | -0.24 | 0.22 | -1.10 | 0.27 |  | |

**p < 0.01; *p </= 0.05

| **Grooming networks** | | | | | |  |
| --- | --- | --- | --- | --- | --- | --- |
| **Predictor** | **B** | **SE** | **Z** | **P** | ***df*** | |
| (Intercept) | -2.20 | 0.38 | -5.82 | <0.01** | 323 | |
| Sex (males vs females) | -0.08 | 0.02 | -4.41 | 0.01* |  | |
| Rank percentile | 0.08 | 0.03 | 2.51 | 0.01* |  | |
| Species (long-tailed vs bonnet) | -0.39 | 0.46 | -0.85 | 0.40 |  | |
| Species (rhesus vs bonnet) | -1.05 | 0.46 | -2.26 | 0.02* |  | |
| Species (long-tailed vs rhesus) | 0.65 | 0.38 | 1.73 | 0.08 |  | |
| Network strength (bonnet) | 0.42 | 0.13 | 3.24 | <0.01** |  | |
| Network strength (rhesus) | 0.53 | 0.10 | 5.45 | <0.01** |  | |
| Network strength (long-tailed) | 0.67 | 0.10 | 6.73 | <0.01** |  | |
| Frequency of interactions with humans | 0.03 | 0.03 | 0.82 | 0.41 |  | |
| Foraging on anthropogenic food | 0.02 | 0.04 | 0.55 | 0.58 |  | |
| Network strength by species (long-tailed vs bonnet) | 0.11 | 0.16 | 0.67 | 0.50 |  | |
| Network strength by species (rhesus vs bonnet) | 0.25 | 0.16 | 1.53 | 0.13 |  | |
| Network strength by species (long-tailed vs rhesus) | -0.14 | 0.14 | -1.01 | 0.31 |  | |

**p < 0.01; *p </= 0.05
